# Investigation on tissue specific effects of pro-apoptotic micro RNAs revealed miR-147b as a potential biomarker in ovarian cancer prognosis

**DOI:** 10.18632/oncotarget.13095

**Published:** 2016-11-04

**Authors:** Michael Kleemann, Jeremias Bereuther, Simon Fischer, Kim Marquart, Simon Hänle, Kristian Unger, Verena Jendrossek, Christian U. Riedel, René Handrick, Kerstin Otte

**Affiliations:** ^1^ Institute of Applied Biotechnology, University of Applied Sciences Biberach, 88400 Biberach, Germany; ^2^ University of Ulm, Faculty of Medicine, 89079 Ulm, Germany; ^3^ Boehringer Ingelheim Pharma GmbH and Co.KG, BP Process Development Germany, 88400 Biberach, Germany; ^4^ Research Unit Radiation Cytogenetics, Helmholtz Zentrum München , 85764 Neuherberg, Germany; ^5^ Institute of Cell Biology (Cancer Research), University of Duisburg-Essen, Medical School, 45122 Essen, Germany

**Keywords:** miR-147b, biomarker, ovarian cancer, apoptosis, prognosis

## Abstract

The development and progression of cancer can be ascribed to imbalances in gene regulation leading to aberrant cellular behavior. The loss of micro RNAs (miRNAs) exhibiting tumor-suppressive function has been demonstrated to be often causative for uncontrolled cell proliferation, migration or tissue infiltration. The installation of de novo tumor suppressive function by using pro-apoptotic miRNAs might be a promising therapeutic approach. In addition, there is a great demand for novel biomarkers for the prognosis of cancer, which prompted us to transfer a high content miRNA screening initially performed to identify bioprocess relevant miRNAs in Chinese hamster ovary (CHO) cells to human cancer cell lines . Analysis of screened miRNAs exhibiting strongest pro-apoptotic effects discovered globally and cross-species active candidates. The recovery rate of apoptosis inducing miRNAs was highest in the human ovarian carcinoma cell line SKOV3. Focusing on ovarian cell lines miR-1912, miR-147b and miR-3073a showed significant apoptosis induction in cell lines with different genetic background (SKOV3^p53null^, OVCAR3^p53R248Q^, TOV21G, TOV112D^p53R175H^, A2780, A2780-cis^p53K351N^) alone and additive effects in combination with carboplatin. While expression analysis revealed a low endogenous expression of miR-1912 and miR-147b in SKOV3, miRNA expression was highly upregulated upon apoptosis induction using chemotherapeutics. Ectopic introduction of these miRNAs lead to enhanced activation of caspase-dependent death signaling and an induction of the pro-apoptotic proteins Bak1 and Bax and a reduced expression of Bcl2 and Bcl-xL. Finally, analysis of The Cancer Genome Atlas data revealed the expression of hsa-miR-147b-5p to show a positive influence on the median survival of ovarian cancer patients.

## INTRODUCTION

Ovarian cancer (ovarian CA) is a common human cancer with dismal prognosis. 2012 the World Health Organization dated 239,000 new cases and 152,000 deaths from ovarian CA. Hence, ovarian CA is the seventh most common cancer in women worldwide and the eighth most common cause of death from cancer [1]. The most frequently used biomarker for ovarian CA diagnosis is Carbohydrate antigen-125 (CA-125) [2]. However sensitivity and specificity of CA-125 is poor as it is only raised in approximately 50% of stage 1 epithelial ovarian CA and in 75–90% of patients with advanced disease [3]. Surgery for staging and debulking followed by a platinum- and taxane-based chemotherapy represent the initial standard treatment regimen (stage I-IVA) [4]. Chemotherapy most commonly employs carboplatin and paclitaxel for 3 to 6 cycles. Paclitaxel inhibits the cell division by binding to α-tubulin and thereby stabilizing the microtubules whereas carboplatin leads to apoptosis induction by DNA cross linking [5] [6]. In recurrent or persistent ovarian CA Bevacizumab (Avastin^®^) can be administered in combination with these first-line drugs to inhibit tumor angiogenesis by binding to vascular epidermal growth factor A (VEGFA) [7].

Apoptosis is considered as altruistic death of single cells without damaging the surrounding tissue [8] and its execution is mainly regulated by two distinct but interrelated signaling cascades, the intrinsic and the extrinsic apoptosis death pathway. The extrinsic pathway involves binding of an pro-apoptotic inductor ligand like Tumor Necrosis Factor Related Apoptosis Inducing Ligand (TRAIL) or Tumor necrosis factor (TNF) to a death receptor [9] whereas the intrinsic pathway is initiated by cytochrome c release from the mitochondria [10]. Apoptosis is executed by a tightly regulated signaling cascade culminating in activation of caspases and subsequent specific morphological and biochemical changes like nuclear condensation, cleavage of genomic DNA and cell shrinkage resulting in programmed cell death and elimination of degenerated cells by phagocytosis [11]. Resistance to apoptosis and other types of cell death is a common feature of cancer cells [12–13].

Micro RNAs (miRNAs) are small non-coding RNAs with a total length of about 19–25 nucleotides [14]. They are highly conserved across species and are encoded in the genome of animals, plants, fungi and virus. MiRNAs are transcribed by RNA-polymerase II and processed by the RNase-III enzymes DROSHA and DICER in the nucleus and cytoplasm, respectively [14]. The resulting single-stranded RNAs are incorporated into the RNA-induced silencing complex (RISC) and guided to specific binding sites on the target messenger RNAs (mRNAs). The miRNA-RISC then induces enhanced degradation by nucleolytic cleavage or translational inhibition of the target mRNAs [14–15]. Due to the imperfect base paring of the miRNA to the mRNA at the target site, miRNAs can target many different genes with overlapping functions resulting in a redundancy in pathway regulation [16].

A variety of miRNAs have been shown to be involved in the regulation of the intrinsic or extrinsic apoptotic pathways by affecting *e.g*. the expression of proteins of the Bcl2-family as well as the expression and activation of caspases or other proto-oncogenes and tumor suppressors [13–17]. The dysregulation of either apoptosis associated genes like Bcl2-family members or of the tumor suppressor p53, controlling Bcl2-family members in a transactivation-dependent (Bax) as well as in transactivation independent (Bcl2, Bcl-xL, Bcl-w, Mcl-1) manner [18–19], may lead to the formation of cancer or treatment resistance [20]. Hence, the modulation of death-inducing miRNA expression may help to overcome apoptosis resistance in cancer cells or influence the outcome in regard to a specific treatment regimen [21] and therefor help to overcome ovarian CA.

In addition, due to their robust expression patterns and stability miRNAs are attractive biomarkers [23] analyzed amongst others for ovarian cancer diagnostics [21].

**Figure 1:**
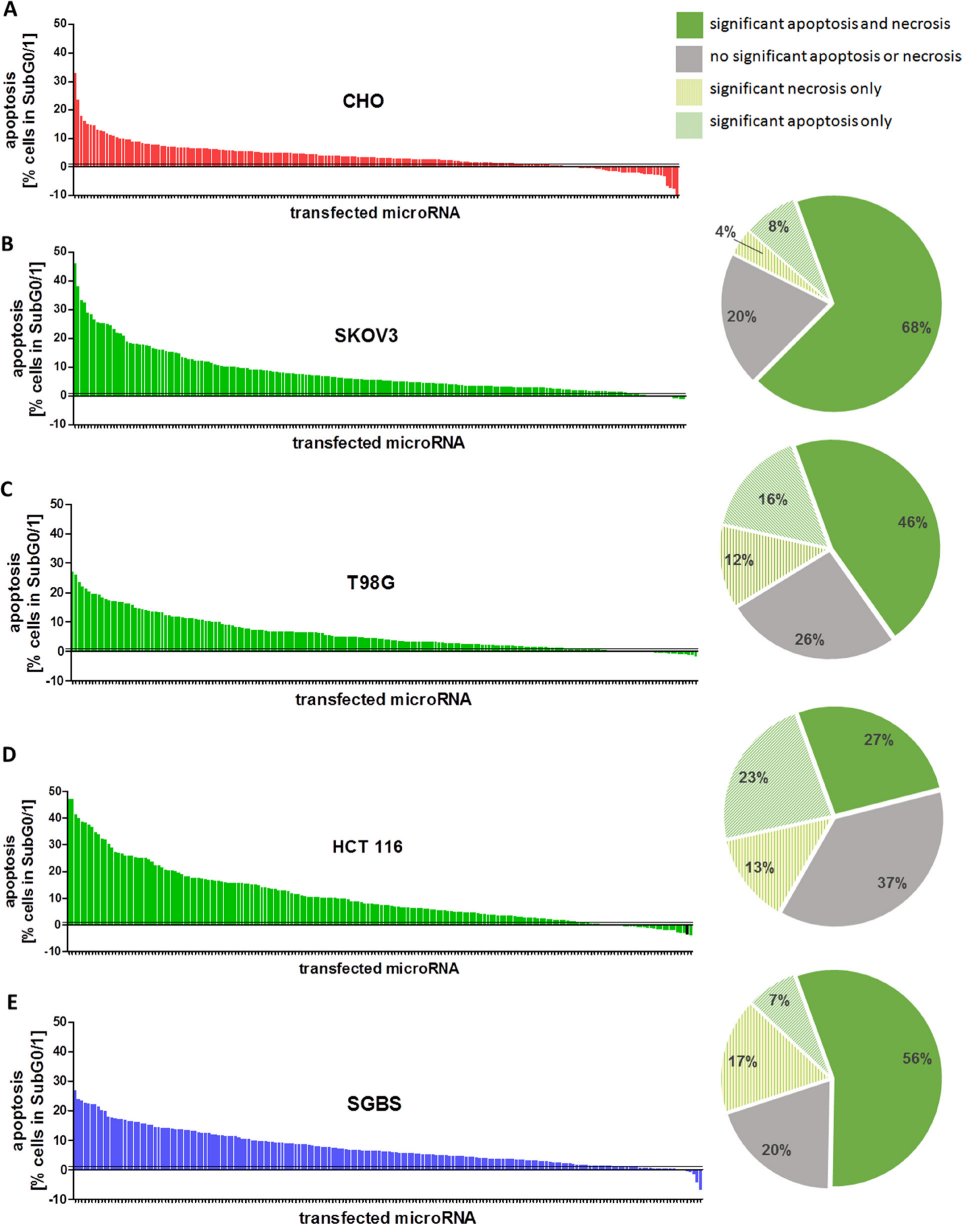
(**A**–**E**) Apoptosis rates after miRNA mimic transfection. Overview of the normalized results of all 188 miRNA mimics selected from the initial screening in CHO cells. The screening was performed in a 96 well plate with a total volume of 100 μl, a final concentration of 50 nM miRNA mimics and 0.4 μl ScreenFect^®^A. The cells were seeded 24 h before transfection and analyzed 72 h after transfection by Nicoletti staining followed by flow cytometry analysis. The mean values [*n* = 3] were normalized by the values of the NT control of the respective cell lines (A) CHO, (B) SKOV3, (C) T98G, (D) HCT 116 and (E) SGBS and arranged from strong to weak apoptotic effect (left panel). The respective numbers of miRNAs which induced significant pro-apoptotic or necrotic effects (*p <* 0.05) are illustrated by cake charts as percentage of the candidates from the miRNA sublibrary tested in total (right panel).

Here we aimed to identify novel miRNAs suited as prognostic biomarkers by validating candidate miRNAs identified in a previous screening in Chinese hamster ovary (CHO) cells [24] in human cancer cell lines. A high-content miRNA screening using a mouse microRNA (mmu-miR) mimic library containing 1,139 different miRNAs, which were individually transfected into a recombinant CHO cell line was performed previously, initially intended to identify bioprocess relevant miRNAs [24]. By examining apoptosis and necrosis using DNA staining assays in combination with flow cytometry analysis we were able to identify a large number of cell death inducing miRNAs [24]. The remarkably high number of impactful miRNAs identified to trigger cell death in CHO cells (Figure 1A) encouraged us to validate these miRNAs in a panel of human cancer cell lines which might give rise to novel target miRNAs for cancer research. A strong induction of apoptosis after miRNA transfection was found in ovarian cancer cell lines with different genetic background. Furthermore, at least sub-additive to additive effects were detected for the potentially effective candidate miRNAs in combination with carboplatin in a larger panel of ovarian CA cell lines. The validation screening identified novel pro-apoptotic miRNAs with potential use as novel therapeutic entities and/or prognostic biomarkers for ovarian cancer.

## RESULTS

### Apoptosis screening in human cancer cell lines

Based on the initial high content screening in CHO cells [24] we created a sub-library consisting of 188 miRNAs identified to induce cell death. Detailed analysis regarding species specificity revealed 106 individual miRNAs (56%) of the sub-library to be mouse specific and therefore not to be expressed in human tissues or even to be absent in the human genome (miRBase Version 21). To ensure a broad diversity of employed cancerous tissues in the sub-screening we selected a glioblastoma cell line (T98G), a colorectal (HCT 116) as well as an ovarian carcinoma cell line (SKOV3). In addition, SGBS preadipocyte cells were employed as a non-immortalized and non-cancerous control cell line for subsequent pro-apoptotic miRNA analyses. After successful adaptation of the previously developed transient miRNA mimics transfection protocol employing ScreenFect^®^A [25], cell type specific screening controls were established by transfecting functional control siRNAs including a non-targeting siRNA (NT), a human cell death inducing siRNA (DT) as well as the two already known pro-apoptotic miRNAs miR-137-3p (T98G, SKOV3, SGBS) and miR-28-5p ( HCT 116 ) as positive controls. In addition, untransfected cells with and without transfection reagent were present on each screening plate. We transiently transfected all 188 pro-apoptotic mmu-miRNAs individually into each of the above mentioned human cancer cell lines in biological triplicates. 72 h post transfection, cells were analyzed for presence of apoptosis and necrosis by quantitative flow cytometry. Furthermore, cell confluence was measured in all wells by automated high-throughput microscopy. The chosen time period of 72 h accounted for both the time-limited transient effects of miRNA mimics and the manifestation of changes in cell phenotype. An increase in specific apoptosis rate was observed in cells transfected with positive control pro-apoptotic miRNAs. The calculated Z-score for NT compared to the respective positive control miRNA ranged from −2.87 to −1.63. The Z-score for NT compared to DT control miRNA transfections ranged between 0.29 and 0.59 (Supplementary Figure S1). These effects were accompanied by a strong decrease in confluency after transfection of death inducing positive control (DT) (Supplementary Figure S2). This was indicative for the functional transfections in all screening plates of all examined cell lines.

To allow for interplate comparisons data normalization was performed by normalizing each sample value to the mean value of the respective on-plate NT-control. In order to see if measured cell death correlates with reduction in cell confluency, the normalized mean confluency values for all 188 miRNAs were plotted against measured apoptotic values. An inverse correlation tendency was observed between cell confluency and cell death represented by apoptosis with correlation coefficient R^2^ up to 0.58 respectively (Supplementary Figure S2). To assess the recovery rate of cell death inducing miRNAs in human cell lines, the normalized mean values considering cell death for all 188 miRNAs were examined individually. Interestingly, the vast majority of the previously identified pro-apoptotic miRNAs in CHO cells were confirmed to induce apoptosis in all four human cell lines tested (Figure 1A–1E). The recovery rate of significantly cell death inducing miRNAs was highest in the human ovarian carcinoma cell line SKOV3 where 150 (80%) of the cell death inducing miRNAs were verified. Of these, 16 miRNAs (9%) induced apoptosis only and 7 miRNAs (4%) induced specifically necrosis. 127 miRNAs (68%) induced significantly both apoptosis and necrosis or late apoptosis. These remarkable results were supplemented by the fact that in human glioblastoma and colorectal carcinoma cells 139 (74%) and 118 (63%) miRNAs significantly increased cell death, respectively, 72 h post transfection (Figure 1B–1E; pie diagram). In the SGBS control cell line, 151 (80%) of the cell death inducing miRNAs were confirmed to induce cell death. Taken together, these high recovery rates of pro-apoptotic miRNAs in a cross-species screening approach argue for an important and possibly conserved role of identified miRNAs in apoptotic pathways.

### Identification of globally and cross species effective miRNAs

**Figure 2:**
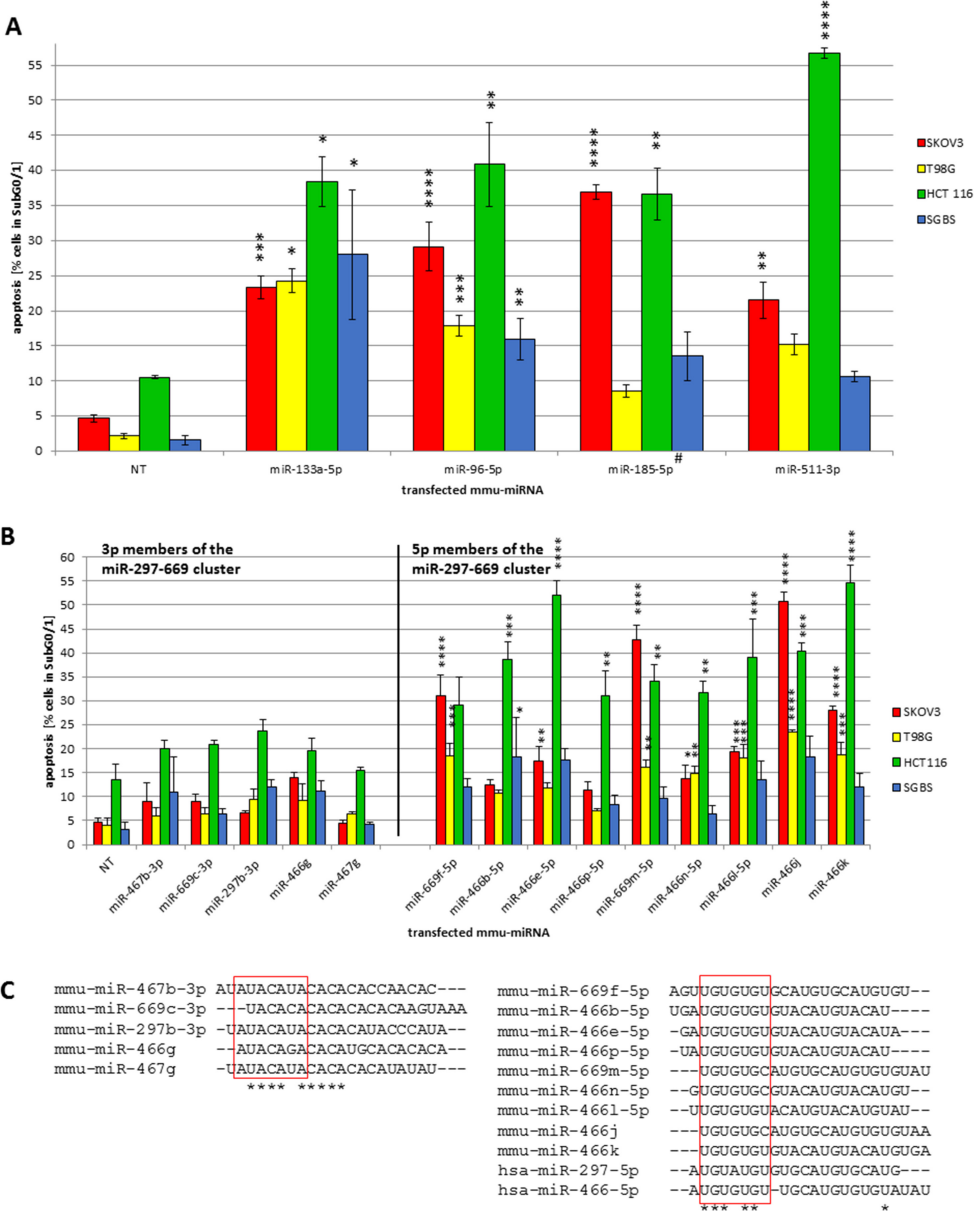
(**A**–**C**) Cross species effects of pro-apoptotic miRNAs - initially identified in CHO [24] - in human tumor cell lines and pro-apoptotic effects of mouse miR-297-669 cluster. The revalidation screening was performed using the conditions described in Figure 1. To compare the effect of the transfected miRNAs (murine, mmu-miR-133a-5p, -96-5p, -185-5p, -551-3p) a non-targeting control (NT) was used (A). The transfected pro-apoptotic miRNA mimics of the 297-669 cluster (B) show a high degree of homology of the putative comparable possible seed sequences for the 3p and the 5p members (C). The sequence homology of the mature miRNAs was analyzed by CLUSTALW. Statistical analysis was performed by two-way ANOVA followed by Bonferroni pos*t-test* [*n* = 3; Mean ± SD, *p < 0.05; **p < 0.01; ***p < 0.001; ****p < 0.0001] #passenger strand (miRBase Version 21).

Due to the large number of discovered cell death inducing miRNAs in the four examined human cell lines we evaluated pro-apoptotic effects in detail and were able to identify miRNAs capable to induce apoptosis in all four cell types. Among these globally effective miRNAs, candidates previously reported to regulate apoptosis in certain human and mouse cell types such as miR-133a-5p, miR-96-5p, miR-185-5p, miR-511-3p [26–29] were identified to induce significant high apoptosis rates (Figure 2A). Highest apoptosis inducing effects were observed for miR-511-3p in HCT 116 cells with 56.7% (± 0.8%). MiR-96-5p and miR-133a-5p induced a significant increase in all four examined cell lines. Hence, miR-96-5p was chosen as a pro-apoptotic control in further experiments. Interestingly, in case of miR-185-5p the passenger strand was identified to significantly induce apoptosis at high rates in SKOV3 (36.9% ± 1.0%) and HCT 116 cells (36.6% ± 3.7%). In summary, these data argue for the validity of the screening procedure employed and identified globally active pro-apoptotic miRNAs.

Since 56% of our sub library was composed of mouse specific miRNAs, we were interested to analyze whether these miRNAs are active across species boundaries in the screened human cell lines. The mouse specific miR-297-669 cluster was previously discovered to induce apoptosis upon transient overexpression in CHO cells [30]. As expected, our previous screening data [24] confirmed most examined members of the mouse miR-297-669 cluster to reliably induce apoptosis in CHO cells. Surprisingly, many members of the mouse specific miR-297-669 cluster were also able to induce apoptosis in the human cancer cell lines SKOV3, T98G and HCT 116 with significantly higher specific apoptosis rates observed for the 5p strands of the miR-466 and miR-669 members (Figure 2B). In HCT 116 cells the highest apoptosis rates were observed for mmu-miR-466e-5p (51.9% ± 3.0%) and mmu-miR-466k (54.7% ± 3.7%). The apoptosis rates in SKOV3 cells were comparable with 42.7% (± 2.9%) for mmu-miR-669m-5p and 50.8% (± 2.0%) for mmu-miR-466j. In contrast, 3p strands of cluster members did not induce apoptosis at significant rates. To understand these effects, we compared seed sequences of all members and found a high degree of homology between putative seed sequences of respective 3p and 5p strands (Figure 2C). Interestingly, the human orthologues of miR-297-5p and miR-466-5p also show similarities in the seed sequence, indicating a potentially cross-species pro-apoptotic function especially for the 5p strands of this miRNA cluster.

### MiRNAs inducing apoptosis in cell line specific manner

**Figure 3:**
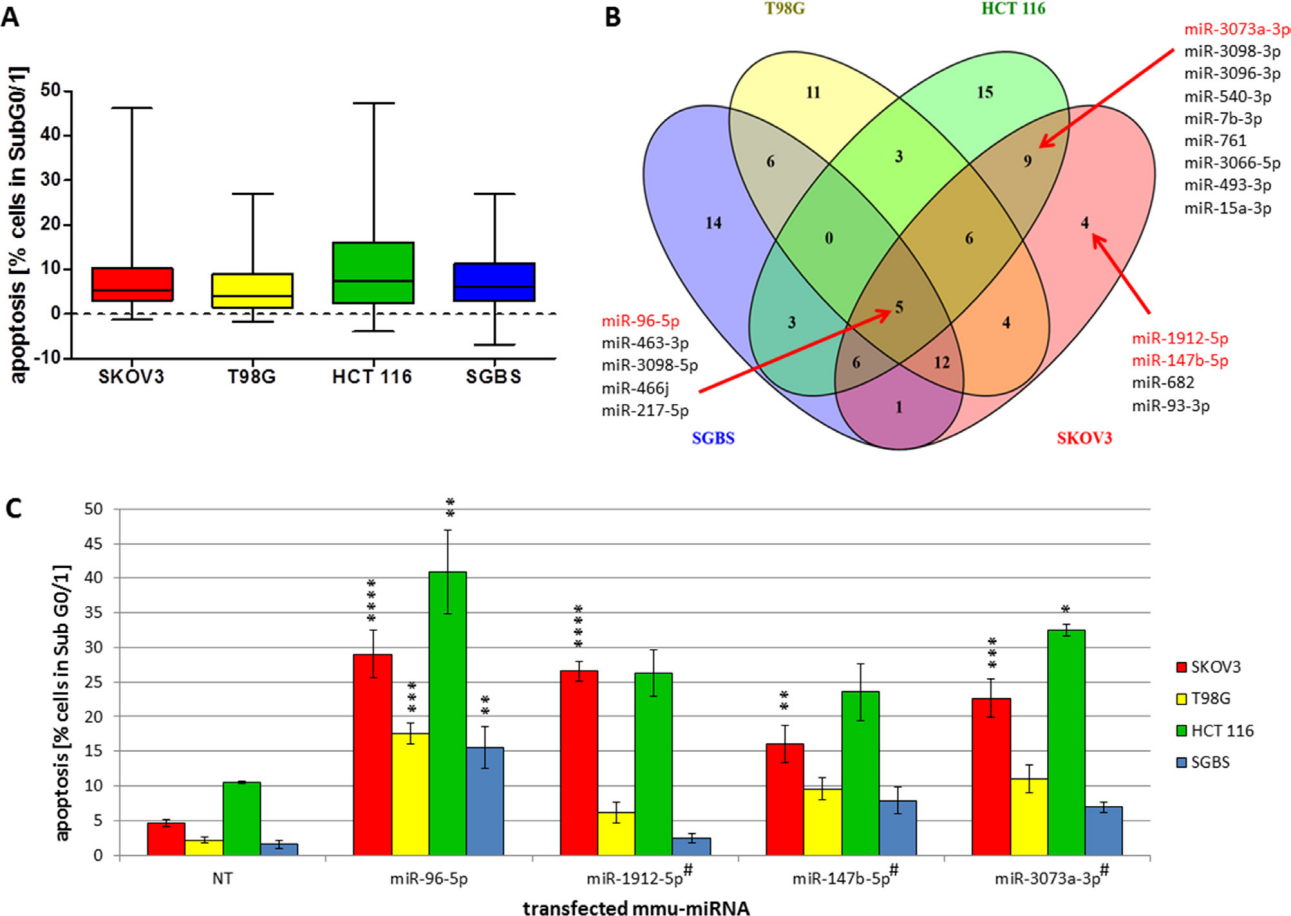
(**A**–**C**) Classification of effect strength and cell specific pro-apoptotic effects of mmu-miR-96-5p, -1912-5p, -147b-5p and -3073a-3p in human cancer cell lines. Quartile classification of the pro-apoptotic effect intensity regarding the specific apoptosis rate of all 188 miRNA mimics selected from the initial screening in CHO cells (conditions of the experiment described in Figure 1). All 188 miRNA were individually transfected in SGBS, SKOV3, T98G and HCT 116 cells. 25% of the values representing the highest relative effects were classified as “strong”. Values with descending effects were classified in “medium”, “weak” or “no” effect, respectively (A). Venn diagram illustrates overlapping as well as cell line specific pro-apoptotic effects of the 47 miRNAs with the strongest pro-apoptotic properties (B). Pro-apoptotic miRNAs marked in red were analyzed in further detail (C). To compare the effects of the transfected miRNAs a non-targeting control (NT) was used. Statistical analysis was performed by two-way ANOVA followed by Bonferroni pos*t-test* [*n* = 3; Mean ± SD, * *p <* 0.05; ** *p <* 0.01; *** *p <* 0.001; ****p < 0.0001]. #passenger strand (miRBase Version 21).

MiRNAs are known to be fine tuners of gene expression and the strength of effects on specific cellular mechanisms including the induction of apoptosis may vary among different tissues [31]. We classified the apoptotic effect intensity of the 188 transfected miRNAs for each examined cell line into quartiles containing 25% of effective miRNAs each (Figure 3A). For all cell lines, half of the miRNAs in quartile 2 and 3 induced apoptosis in 2% to 16% of transfected cells. MiRNAs displaying the strongest apoptosis induction showed a wide spreading of effect strength with up to 47% of apoptosis in HCT 116 cells and 46% of apoptosis in SKOV3 cells.

It has previously been shown that miRNAs display divergent effects depending on cellular contexts, where some miRNAs induce apoptosis in a cell line specific manner and other miRNAs seem to have overlapping pro-apoptotic functions in different cell lines [15]. In this context, we selected the 47 miRNAs found to induce the strongest apoptotic effect clustered in the 4^th^ quartile and examined them for both overlapping and cell line specific pro-apoptotic functions in all four human cell lines. As expected, both cell line specific as well as globally active pro-apoptotic miRNAs were identified (Figure 3B). We identified miR-96-5p, -217-5p, -463-3p, -466j, and -3098-5p to globally induce apoptosis in all cell lines examined. In addition, cell line specific pro-apoptotic miRNAs were identified with 11, 15 or 14 miRNAs inducing apoptosis exclusively in T98G, HCT 116 or SGBS cells, respectively. Surprisingly only a few miRNAs including miR-1912-5p, -147b-5p, -682 and -93-3p were identified to induce apoptosis specifically in SKOV3 cells. Of these miR-1912, miR-147b and miR-93 are expressed in human tissues while miR-682 was found to be mouse specific (miRBase Version 21).

Considering the apparent resemblance to the originally screened CHO cell line and the high recovery rate of apoptosis-inducing miRNAs (Figure 1B), we focused on further detailed analysis of pro-apoptotic miRNAs in the ovarian carcinoma cell lines. Hence, we selected the pro-apoptotic miR-147b-5p and miR-1912-5p showing a strong apoptosis induction in SKOV3 cells in addition to one mouse specific miRNA (miR-3073a-3p) showing pro-apoptotic effects in HCT 116 and SKOV3 cells. MiR-96-5p was identified as a potentially globally active miRNA in all four cell lines (Figure 3B). The results of the apoptosis analysis for all four miRNAs are shown in Figure 3C, where miR-96-5p indeed induced significantly high apoptosis rates in all cell lines when compared to the NT control. In contrast, miR-1912-5p and miR-147b-5p induced significantly high apoptosis rates only in SKOV3 cells relative to NT with 22.1% (± 1.4%) and 11.9% (± 2.8%) 72 h after transfection, whereas the effects in T98G, HCT 116 and SGBS were not significant compared to the control. The mouse specific miR-3073a-3p induced apoptosis in both SKOV3 and HCT-116 cells with 17.4% (± 1.8%) and 37.4% (± 3.6%), respectively. Based on these data, we further focused on the effects of the potentially SKOV3 specific miR-1912-5p and miR-147b-5p.

### miR-1912-5p, miR-147b-5p, miR-96-5p and miR-3073a-3p mediate pro-apoptotic effects in various ovarian cancer cell lines

In order to substantiate our observations on pro-apoptotic effects of miR-1912-5p, miR-147b-5p, miR-96-5p and miR-3073a-3p in ovarian cell lines and to provide a better understanding of the underlying molecular mechanisms of action, further ovarian cancer cell lines with different genetic backgrounds were tested. These included besides SKOV3^p53null^ the highly invasive cell line OVCAR3^p53R248Q^ [32], the cell lines TOV21G and TOV112D^p53R175H^, with the latter expressing non-functional p53 protein with a mutation in the DNA-binding domain [33] as well as A2780-cis^p53K351N^ and A2780 with and without carboplatin resistance, respectively [34–35].

**Figure 4:**
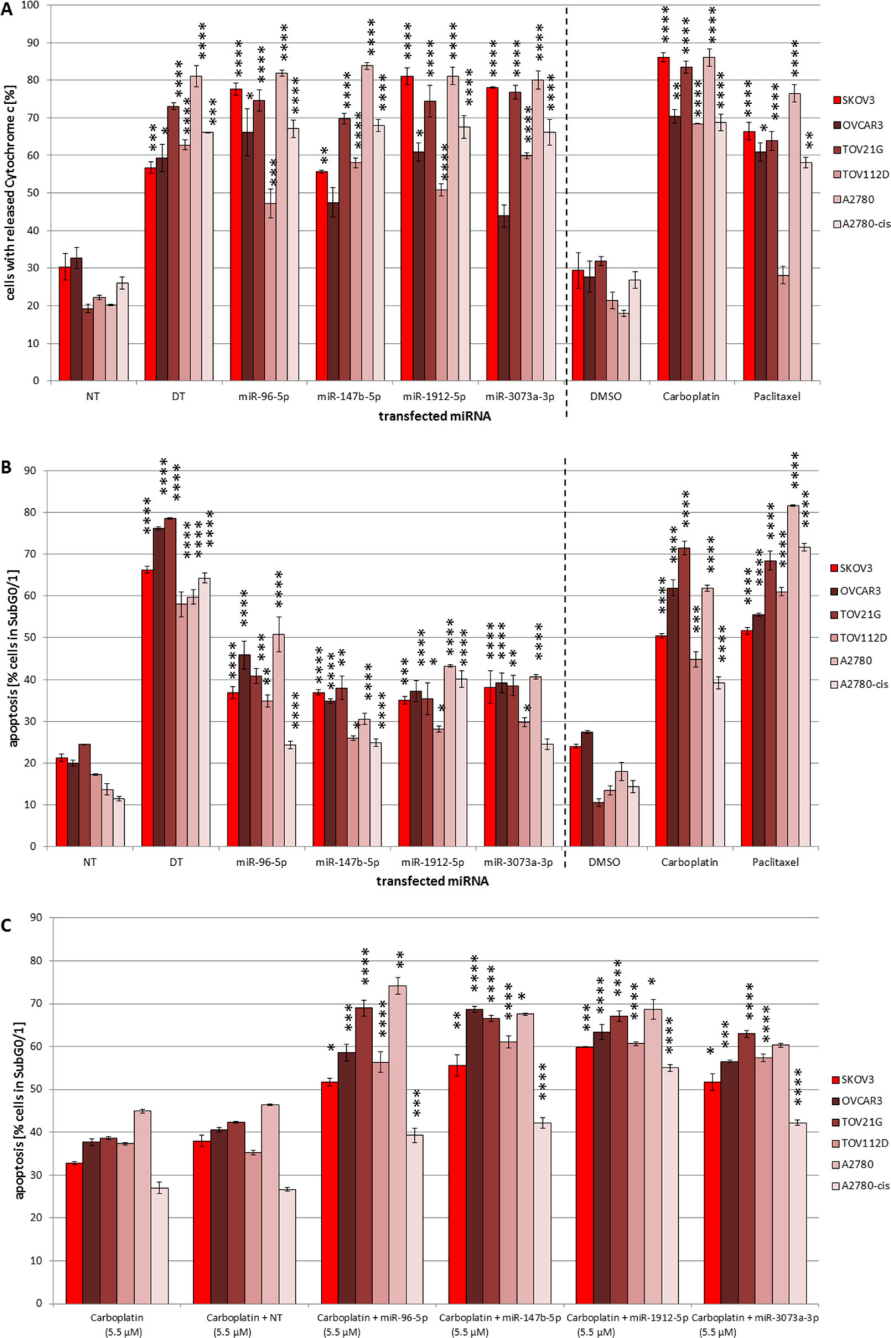
(**A**–**C**) Pro-apoptotic effects of mmu-miR-96-5p, -1912-5p, -147b-5p and -3073a-3p in ovarian cancer cell lines and combined action with carboplatin. Influence of mmu-miR-96-5p, -1912-5p, -147b-5p and -3073a-3p on cytochrome c release (A) and apoptosis rate (B). The miRNA mimics were individually transfected into SKOV3, OVCAR3, TOV21G, TOV112D, A2780 and A2780-cis cells (conditions of the experiment as described in Figure 1). For controls cells were treated with either 8 μM carboplatin or 0.25 nM paclitaxel. Synergistic pro-apoptotic effects of transfected miRNA mimics together the chemo drug carboplatin (C). 5.5 μM carboplatin were added 6 h after transfection. To compare the effect of the transfected miRNAs a non-targeting control (NT) was used. Statistical analysis was performed by two-way ANOVA followed by Bonferroni pos*t-test* [*n* = 3; Mean ± SD, *p < 0.05; **p < 0.01; ***p < 0.001; ****p < 0.0001].

Pro-apoptotic effects were verified for all four miRNAs tested in SKOV3, OVCAR3, TOV21G and A2780 cells in the range of 30.5% (± 1.3%) to 50.8% (± 4.1%) apoptosis at the level of DNA-fragmentation independent off the genetic background (Figure 4B). However, the TOV112D cells expressing a mutant p53 showed lower or less significant apoptosis induction respectively. Interestingly, apoptosis induction by miR-1912-5p and miR-147b-5p in cisplatin resistant A2780-cis cells (40.1% ± 1.9% vs. 24.9% ± 1.0%) was almost comparable to the parental A2780 cells (43.3% ± 0.3% vs. 30.5% ± 1.3%), with a slightly stronger effect of miR-1912-5p in both cell lines. MiR-3073a-3p and miR-96-5p also showed an increase in the subG0/G1 population of drug-resistant A2780-cis almost comparable to the apoptosis rate caused by miR147-5p. However, the effects of miR-3073a-3p and miR-96-5p in the parental line A2780 were even 65% to 109% higher than in A2780-cis cells.

For the chemotherapeutic drugs carboplatin and paclitaxel used as control apoptosis rates of 40% to 80% were detected.

To gain insight into the underlying mechanisms, further analyses were performed to detect alterations in mitochondrial membrane potential (ΔΨm) by flow cytometry employing the potential-sensitive fluorescent dye tetramethylrhodamine ethyl ester (TMRE) and to measure the release of cytochrome c (Figure 4A).

A strong increase in the amount of cells with low ΔΨm in the presence of miRNA mimics was detectable within 48 h post transfection (SKOV3, miR-147b-5p, 54.0% ± 3.0% followed by almost comparable effects of miR-3073a-5p, 48.7% ± 3.2%, miR-96-5p, 47.4% ± 1.4% and miR-1912-5p, 40.0% ± 2.1%; Figure 6C).

**Figure 5:**
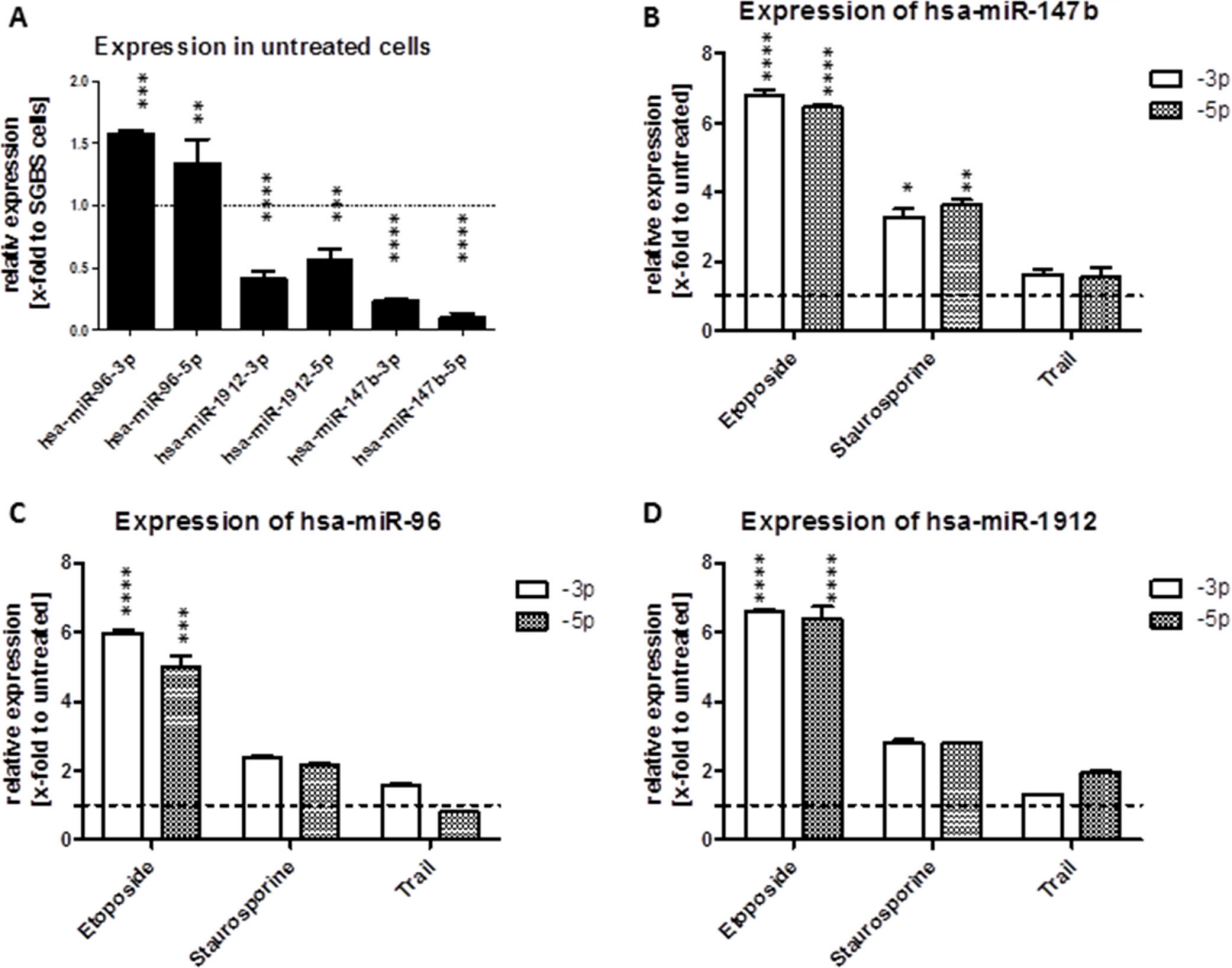
(**A**–**D**) Modulation of miRNA expression after induction of apoptosis. Effects of apoptosis inducers on the expression of mature hsa-miR-96 (C), hsa-miR-147b (B) and hsa-miR-1912 (D) in SKOV3 cells. The cells were seeded in a 6-Wellplate 24 h before treatment. 48 h after treatment with 25 nM staurosporine, 25 μM etopodise or 15 ng/μl TRAIL the cells were harvested for total RNA isolation followed by qRT-PCR. (A) Basal expression levels of mature miRNAs in SKOV3 cells were normalized to relative expression in SGBS cells (relative to an internal U6 control). The expression levels of the mature miRNAs in SKOV3 and SGBS cells was measured 48 h after treatment with the apoptosis inductors staurosporine, etoposide or TRAIL (relative to an internal U6 control and to the untreated cells of the according cell line).

**Figure 6:**
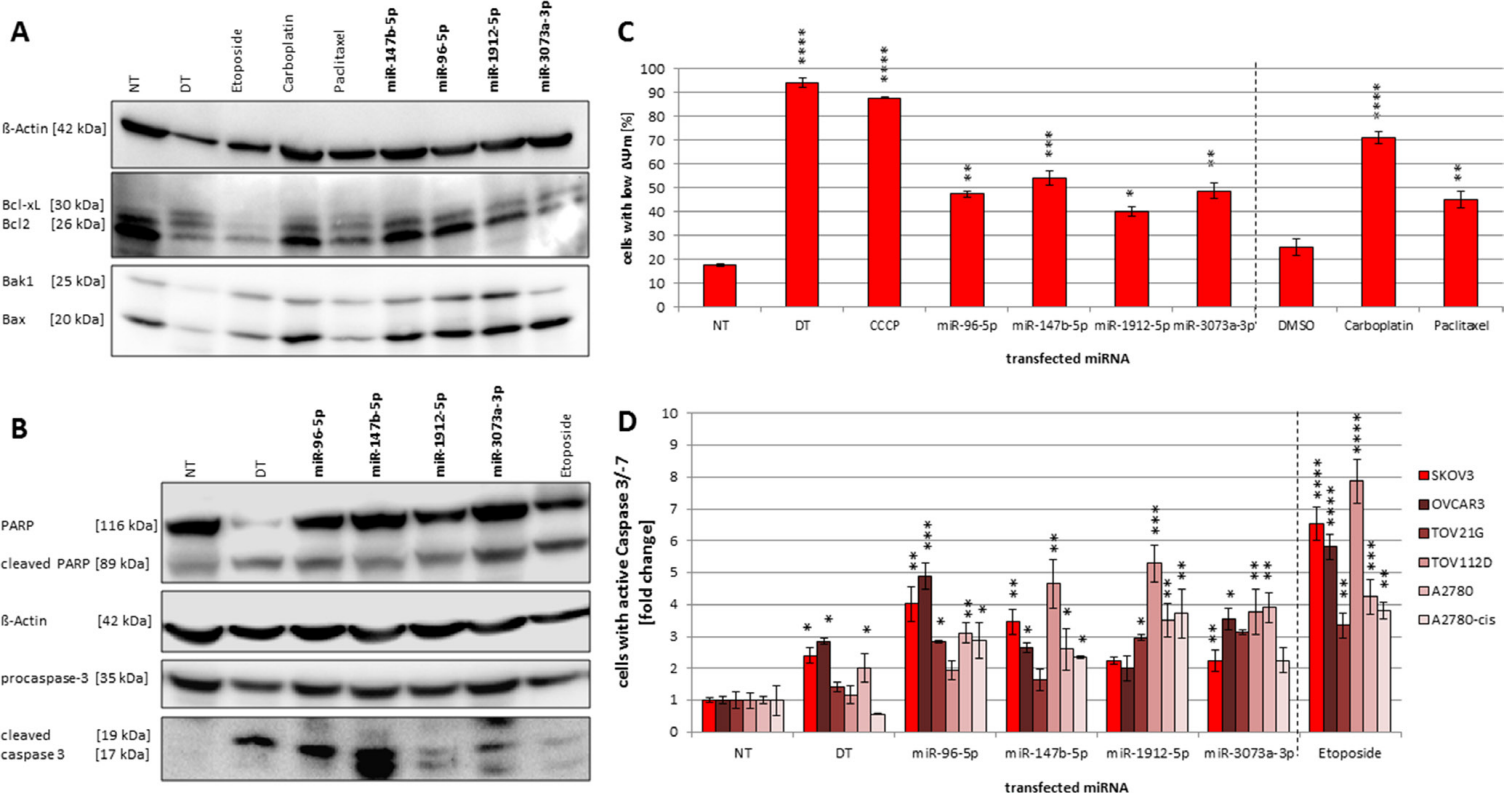
(**A**–**D**) Influences on Bcl2 family members as well as mitochondrial potential and caspase-3 activity. Immunoblot detecting Bcl2, Bcl-xL, Bak1 and Bax (A) as well as procaspase-3 and PARP cleavage (B) after miRNA transfection. SKOV3 cells were seeded in a 96-Wellplate with a total volume of 100 μl 24 h before treatment. 25 μM etoposide, 8 μM carboplatin or 0.25 nM paclitaxel were used as a positive control as well as a transfection with a functional death control siRNA (DT). A non-targeting miRNA (NT) was used as negative control. 72 h after transfection with a final concentration of 50 nM miRNA mimics and 0.4 μl ScreenFect^®^A cells were harvested followed by SDS-PAGE and Immunoblotting with the respective antibodies. To correlate the blot intensity ß-Actin was used as loading control. The densitometry analyses were done using Fusion Software. Influence of mmu-miR-96-5p, -1912-5p, -147b-5p and -3073a-3p on mitochondrial potential (ΔΨm) in SKOV3 cells (C) and caspase-3/−7 activity in ovarian cancer cell lines (D). The miRNA mimics were individually transfected into SKOV3, OVCAR3, TOV21G, TOV112D, A2780 and A2780-cis cells (conditions of the experiment as described in Figure 1). For controls cells were treated with either 8 μM carboplatin or 0.25 nM paclitaxel. To compare the effect of the transfected miRNAs a non-targeting control (NT) was used. Statistical analysis was performed by two-way ANOVA followed by Bonferroni pos*t-test* [*n* = 3; Mean ± SD, *p < 0.05; **p < 0.01; ***p < 0.001; ****p < 0.0001].

Highly significant changes in cytoplasmic levels of cytochrome c compared to the NT control were observed for SKOV3, TOV21G and A2780. However, cytochrome c release was less prominent in OVCAR3 cells and in both the p53 mutated TOV112D cell line as well as in the cisplatin resistant A2780-cis (Figure 4A), pointing towards an potential involvement of the intrinsic apoptosis pathway for all miRNAs in most cell lines.

### Effects of combined treatment with carboplatin

In order to analyze potential synergistic effects with drugs already in use for ovarian cancer treatment, we analyzed apoptosis induction by carboplatin in combination with miR-1912-5p, -147b-5p, -96-5p or -3073a-3p in the established ovarian cancer cell line panel. Determination of apoptotic DNA fragmentation showed significant sub-additive to additive effects in all ovarian cell lines employing 5.5 μM carboplatin alone compared to carboplatin in combination with NT-control (Figure 4C), with highest effects observed in SKOV3, OVCAR3, TOV21G and A2780. Even TOV112D and the cisplatin resistant cell line A2780-cis showed increased apoptosis induction upon combined treatment. Compared to carboplatin in combination with NT-control the combination of carboplatin with miR-147b-5p increased the apoptotic ratio in the cisplatin resistant cells significantly to 42.2% (± 1.3%). In case of A2780-cis the strongest increase was mediated by miR-1912-5p. While 5.5 μM carboplatin in combination with NT resulted in 26.7% (± 0.5%) apoptosis the number of cells in subG0/G1 was almost doubled to 55.0% (± 0.8%) by the addition of 50 nM miR-1912-5p.

### Molecular changes during miRNA induced apoptosis

The induction of apoptosis can be triggered via different molecular pathways. The regulation of apoptosis as well as the expression of miRNAs in cancer cells was reported to be often deregulated [36]. Hence we analyzed the basal expression of both strands of the three human miRNAs miR-96, miR-1912 and miR-147b in SKOV3 cells and observed an upregulation of both mature hsa-miR-96 strands in SKOV3 cells compared to the non-cancerous cell line SGBS. However, hsa-miR-1912-5p and -3p showed decreased expression (0.5-fold) and miR-147b abundance was even further decreased (0.2-fold) compared to SGBS cells (Figure 5A).

To examine the effect of mitochondrial and receptor mediated apoptosis induction on the expression of all three miRNAs, we triggered apoptosis in SKOV3 and SGBS cells using 20 μM etoposide, 25 nM staurosporine or 20 ng/μl TRAIL, which are well known apoptosis inducers. The expression of the 5-prime and 3-prime strand of hsa-miR-96, -1912 and -147b was analyzed 48 h after treatment by qRT-PCR. For miR-96, induction of apoptosis using etoposide led to an enhanced expression of both mature miRNA strands in both cell lines tested, whereas apoptosis induction via staurosporine in SKOV3 cells only induced elevated expression of miR-96. Apoptosis induction using TRAIL led to a slight induction of hsa-miR-96-3p only. For hsa-miR-1912 an upregulated miRNA expression using all three apoptosis inductors was observed. The highest upregulation of the hsa-miR-1912 level was measured after etoposide treatment with an increase of about 6.4 (± 0.3)-fold. Cellular expression of hsa-miR-147b was clearly increased after treatment with all three apoptosis inductors in SKOV3 cells. After etoposide treatment expression of both strands of miR-147b was increased by 6.5-fold (*p <* 0.001), after staurosporine treatment by 3.3-fold and only 1.6-fold (*p >* 0.05) after treatment with TRAIL (Figure 5B–5D).

The observed upregulation of miRNA expression after treatment with apoptosis inducing agents points towards an involvement of the analyzed miRNAs in the initiation or progression of apoptosis in the examined cell lines. In order to investigate possible molecular mechanisms underlying these apoptosis inducing effects, we examined the influence of the selected miRNAs on molecular downstream effectors of mitochondrial apoptosis including pro- and anti-apoptotic Bcl2 family members as well as the apoptosis downstream effectors caspase-3 and PARP. SKOV3 cells were transiently transfected with all above analyzed miRNAs (miR-96-5p, miR-147b-5p, miR-1912-5p and miR-3073a-3p), harvested 72 h post transfection and subjected to immunoblotting. In parallel, cells were treated with etoposide as a positive control for apoptosis induction. After ectopic introduction of miR-96, miR-1912 and miR-147b we detected an enhanced expression of pro-apoptotic proteins Bak1 and Bax for all miRNAs except the mouse-specific miR-3073a, which only showed elevated expression of Bax (Figure 6A). In contrast, the expression of anti-apoptotic proteins Bcl2 and Bcl-xl was decreased after transfection with all miRNAs compared to NT control. Analyzing the downstream effectors of apoptosis, we observed a three-fold increase of cleavaged caspase-3 (p17/p19) abundance and a moderate decrease in procaspase-3 for miR-96, miR-1912 and miR-3073a, while miR-147b even induced a sevenfold increase in caspase-3. Transient introduction of all four miRNA mimics individually induced moderate activation of PARP cleavage between 1.2 to 1.7-fold (Figure 6B). Using the caspase activity assay for detection of active caspase-3/−7 an increase of 4.0-fold after miR-96 transfection and 3.5-fold increase after miR-147 transfection in SKOV3 cells was detected (Figure 6D).

These data support our previous findings that miR-3073a, miR-1912 and miR-147b seem to be involved in the progress of apoptosis in ovarian carcinoma cells and induce molecular changes including proteins of the intrinsic pathway as well as downstream effectors of apoptosis.

### miR-147b as potential biomarker for ovarian cancer

**Figure 7:**
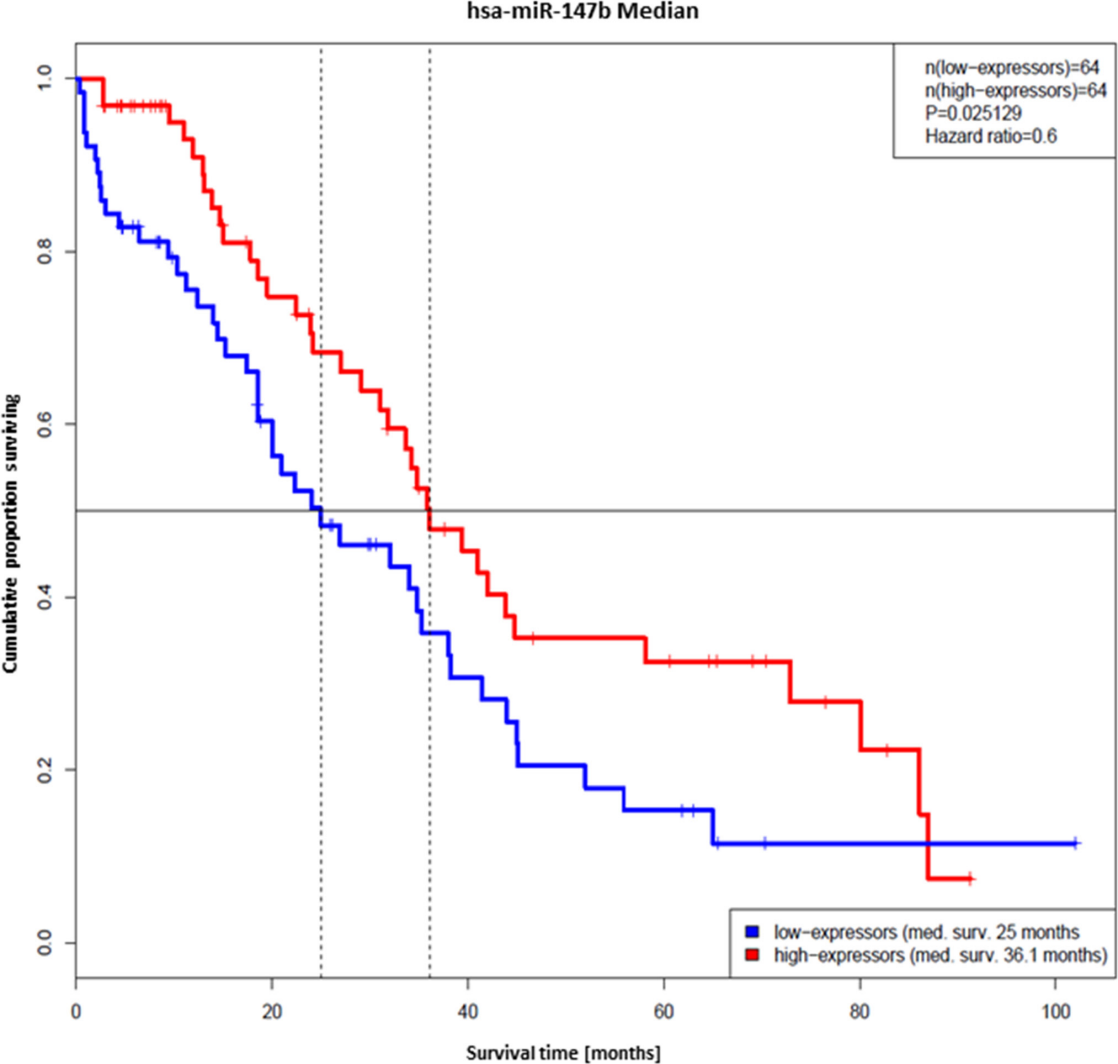
Hsa-miR-147b-5p as biomarker for the median survival of ovarian cancer patients. Prolonged survival time of ovarian cancer patients expressing hsa-miR-147b-5p. Kaplan-Meier plots of the groups with tumors expressing hsa-miR-147b-5p at low (blue) and high (red) levels. The median survival time of the low-expressors was 25 months and that of the high-expressors 36.1 months.

There is still a high need for the definition of prognostic biomarkers for cancer patients. In order to characterize the impact of the identified pro-apoptotic miRNAs on median survival of ovarian cancer patients, miRNA expression data of the “Ovarian serous cystadenocarcinoma” data set were analyzed with clinical data from the TCGA data base [37]. Mean age of patients was 60.2 years. The analysis detected a statistical significant impact of one of the above analyzed miRNA, hsa-miR-147b, on median survival in patients older than 69 years (3^rd^ quartile, *n* = 129). In this group patients with tumors expressing hsa-miR-147b-5p at high- and low-levels the miRNA was suggested to have a protective effect on survival (Hazard-ratio: 0.6, 95%-confidence interval: 0.38 - 0.94, *p-value*: 0.025). The median survival of the low-expressor group was 25 months and that of the high-expressors 36.1 months (Figure 7). Patients with elevated miR-147b-5p levels exhibited a lower risk of death within the observed time frame. Hence, miR-147b-5p expression might be considered as a protective prognostic marker in ovarian cancer in this cohort.

## DISCUSSION

In most cases, the development and progression of cancer can be ascribed to imbalances in gene regulation leading to aberrant cellular behavior [12]. MiRNAs play an essential role in fine-tuning gene expression and the loss of miRNAs exhibiting tumor-suppressive function has been demonstrated to be often causative for uncontrolled cell proliferation, migration or tissue infiltration [38–40]. The reconstitution of endogenous expression of tumor suppressive miRNAs or the installation of de novo tumor suppressive function by using pro-apoptotic miRNAs might be a promising therapeutic approach to expand the list of potential miRNA therapeutics in humans. Apart from that, miRNA signatures in both tissue and body fluids would allow for the diagnosis and prognosis of severe diseases as well as for the selection of appropriate treatment regime [41–42]. In this regard, pro-apoptotic miRNAs might be attractive novel biomarker candidates in the context of cancer diagnostics, prognosis and treatment selection.

In order to identify novel pro-apoptotic miRNAs for their potential suitability as cancer therapeutics or prognostic biomarkers we transferred an initial high content miRNA screening of murine miRNAs in CHO cells [24] to human cancer cell lines.

The functionality of the screening procedure was assured by using the pro-apoptotic miRNAs miR-137-3p and miR-28-5p as functional controls [43, 44]. Both miRNAs showed an upregulation of apoptosis compared to NT controls. The Z-score of up to 0.59 for the comparison of the human cell death control siRNA to NT control supported the reliability of the data [45]. The observed increase in apoptosis rates was accompanied by a decrease in cell confluency emphasizing the phenotypical relevance of cellular effects.

Interestingly, the majority of the previously identified pro-apoptotic miRNAs was confirmed to induce cell death in all four human cancer cell lines tested. As hypothesized, the recovery rate of apoptosis inducing miRNAs was highest in the human ovarian carcinoma cell line SKOV3, where effects of 80% of the pro-apoptotic miRNAs were verified. These remarkable recovery rate might support an evolutionary conservation of molecular pathways for the process of apoptosis [46] and the presence of highly conserved cell death associated miRNA to target gene relationships in mammalian cells. However, the results revealed differences in the number of pro-apoptotic miRNA hits between the different cell types with lower recovery rates observed for the colorectal cancer cell line HCT116 (62%) and the glioblastoma cell line T98G (49%). MicroRNAs that modulate global gene expression in eukaryotic cells at the post-transcriptional level are highly conserved across species [47]. However, several studies demonstrate their tissue specific expression and function [48] and, although the apoptotic pathways might be conserved across species, the stimulus to trigger apoptosis might be cell type dependent [11].

Analysis of miRNAs exhibiting strongest apoptotic effects in SKOV3, HCT 116, T98G and SGBS discovered globally active candidates inducing apoptosis in all cell types analyzed. Among those, miR-96 is a well characterized cell death inducing miRNA showing pro-apoptotic effects *e.g*. in non-small cell lung cancer (NSCLC) [49]. It is involved in the development of different kind of cancers like bladder cancer [50], hepatocellular carcinoma [51] or esophageal cancer [52]. The further identified miR-133a has been shown to reduce cell viability by inducing apoptosis in the OVCAR3 ovarian cancer cell line and was therefore suggested as a potential biomarker for the prediction of epithelial ovarian cancer pathogenesis and progression [53].

Due to the fact that the initial screening was performed in CHO cells using a mouse genome wide miRNA mimic library, analysis of the sub-library of 188 significantly effective pro-apoptotic miRNAs identified more than half of the miRNAs (56%) to be mouse specific and either not to be expressed in human cells or even not to be present on the human genome. Hence, we were interested whether these mouse specific miRNAs were active across species boundaries in the screened human cell lines. In depth analysis identified the mouse specific miR-297-669 cluster previously discovered to induce apoptosis upon nutrient depletion in CHO cells [54]. This cluster is composed of 28 miRNAs located in intron 10 of the mouse Sfmbt2 gene on chromosome 2. The sole human orthologues are hsa-miR-466 located in an intergenic region on chromosome 3 and hsa-miR-297, a highly cytotoxic microRNA in glioblastoma associated with reduced *in vitro* invasiveness and *in vivo* tumor formation, located on chromosome 4 [55]. Surprisingly, the analyzed miRNAs of the entire mouse specific miR-297-669 cluster showed significantly high apoptosis rates in the human tumor cell lines analyzed, especially after transfection of the 5p members. The conservation of seed sequences of all 5p strands indicates that sequence homology might play an important role in the induction of apoptosis by recognizing potentially conserved mRNA targets across species. Such conservation has been previously shown for miRNA seeds in mammals [56] and subsequent conservation of function may might be indicated by the only human orthologue hsa-miR-297 which has been shown to induce apoptosis in glioblastoma cells [55]. The murine specific mmu-miR-3073a-3p was identified to induce strong apoptotic effects in the human cancer cell lines HCT 116 and all ovarian cell lines tested. This miRNA is encoded on the murine chromosome 12 in the protein coding sequence of asparaginase. There are no validated mRNA targets known for miR-3073a so far. As the use of tumor suppressive miRNAs for cancer therapy has been shown to be an attractive strategy [57], the installation of a de novo tumor suppressive function employing pro-apoptotic miRNAs from other species, *e.g*. mouse, which are not encoded by the human genome, might be a promising approach to expand the list of potential miRNA-based therapeutics in humans. Due to the fact that human cells do not express these miRNAs endogenously, the presence of the ectopically introduced miRNAs, *e.g*. using viral vectors may be monitored and controlled more precisely.

Tissue specificity of miRNA effects might be based on the coexpression with corresponding miRNA regulators [57]. These miRNAs may therefore represent attractive targets for tissue specific tumor therapy.MiR- 147b-5p and miR-1912-5p showed significant pro-apoptotic effects in ovarian SKOV3p53null cells. These miRNAs are conserved between mouse and human and are located on chromosomes 15 and X, respectively. Significant pro-apoptotic effects of miR-96-5p, -147b-5p, -1912-5p and mmu-miR-3073a-3p were also found in OVCAR3^p53R248Q^, TOV21G and TOV112D^p53R175H^, A2780 and point to a death inducing potential of the discovered miRNAs in ovarian cancer cells in general and at least partially independent on the genetic background of the cells. Surprisingly, the identified subpanel of miRNAs was able to induce apoptosis even in the cisplatin resistant cell line A2780-cis^p53K351N^ with miR-1912 being most efficient in reconstitution of apoptotic response compared to the parental A2780. Considering mutations of p53, upregulated DNA repair mechanisms as well as upregulation of ABC transporter family efflux pumps may be responsible at least in part for primary resistance to DNA damage induced by carboplatin [34–35]. MiR-1912 might overcome these resistance mechanisms by p53 independent mode of action and might open a novel therapeutic option in regard to potential targeted treatment of frequent recurrent platinum-resistant ovarian cancer [58].

Assuming the high recovery rates of pro-apoptotic miRNAs we focused on the molecular mechanisms of tissue specific miRNA in SKOV3 ovarian carcinoma cell lines. The further analyses of mechanisms underlying the process of apoptosis demonstrated that transient overexpression of miR-147b as well as miR-96-5p, -1912-5p and -3073a-3p led to an enhanced activation of caspase-dependent death signaling in ovarian cancer cell lines. The inversely altered expression patterns of pro-apoptotic proteins Bax/Bak compared to the anti-apoptotic proteins Bcl2 and Bcl-xL, concordant with the elevated cytoplasmic cytochrome c levels and early breakdown of mitochondrial membrane potential, suggest for the involvement of mitochondrial apoptosis signaling in response to miR-147b-5p, miR-1912-5p, miR-96-5p and miR-3073a-3p resulting in the activation of caspase-3 and -7 and inactivation of PARP. There are several miRNAs known to target mitochondrial death pathway directly or indirectly and some of them are known to modulate Bcl2-family members. The expression of Bcl2 has been shown to be down-regulated after treatment with miR-206 [59]. Conversely, up-regulation of Bcl2 was confirmed in human lung cancer tissue samples, inversely correlated with miR-206 expression and validated miR-206 binding sites are present within the 3′-untranslated region (3′-UTR) of Bcl2 [59]. In Chronic lymphocytic leukemia patients Bcl2 expression is inversely correlated with miR-15a and miR-16-1 and both microRNAs negatively regulate Bcl2 at the posttranscriptional level [60]. Expression of Bcl-xL is shown to be suppressed by miR-377 recognizing two functional binding sites in the Bcl-xL 3′-UTR [61]. On the other hand, for the oncomiR miR-150, that is overexpressed in human NSCLC, a direct interaction with pro-apoptotic Bak leading to inhibition of cell proliferation and apoptosis has been shown [62]. However, referring to in silico prediction (microRNA. org, [63]) no potential binding sites for miR-96, -147b or -1912 are present on known Bcl2-family mRNAs. Thus, the observed regulatory effect at the level of Bcl2 proteins might be mediated by a modulation upstream or downstream of the corresponding signaling cascades.

Expression of pro-apoptotic Bcl2-family members is controlled by tumor suppressor p53 that exhibit transcriptional dependent and independent modes of action and induces *e.g*. the trans-activation of Bax, trans-repression of anti-apoptotic Bcl-2 or survivin or directly binds to Bak inducing its oligomerization at the mitochondria, followed by cytochrome c release [56, 64]. As shown by Feng et al., p53 modifies the transcription of different miRNAs and is itself regulated by other miRNAs [65–66]. p53 and MDM4, a strong p53 transactivation inhibitor, are direct targets of tumor suppressor miR-34a (MRX34) [67]. It is also known that miR-34 lead to apoptosis by modulation of Bcl2 levels [66]. However, tumor suppressor p53 is frequently mutated or deleted in high-grade ovarian cancer. With respect to the p53 state of the ovarian cancer cell lines with different genetic background harboring either wildtype (TOV21G, A2780) or functionally mutated p53 (OVCAR3^p53R248Q^, TOV112D^p53R175H^, A2780-cis^p53K351N^), or even lacking p53 expression (SKOV3) the strong pro-apoptotic effects observed in the recent study might suggest a potential p53-independent mode of action of miR-1912-5p, -147b-5p, -96-5p and -3073a-3p that offers the opportunity to overcome p53 dependent treatment resistance.

The tight regulation of apoptosis is essential for the cell, however, regulation of apoptosis [17] as well as expression of miRNAs are frequently deregulated in cancer cells [36]. The analysis of basal expression of specific pro-apoptotic miRNAs miR-147b and miR-1912 in the ovarian cancer cells compared to non-cancerous SGBS cells demonstrated a reduced expression in ovarian cancer cell lines. This is in line with previous data showing that the expression of miR-147b and miR-1912 is also downregulated in other kinds of cancer tissues like breast cancer or non-small cell lung cancer compared to non-cancerous tissue [68, 69]. Surprisingly, the miR-147b level was further shown to be influenced by chemotherapeutic drugs such as staurosporine or etoposide forcing cancer cells into apoptosis via intrinsic signaling pathway. After induction of apoptosis especially the expression of miR-147b resulted in a two to seven-fold upregulation depending on the apoptosis inductor applied. The changes in the miRNA expression may lead to a different behavior of the cell with an altered regulation of their death pathways [17] [70].

Besides request for novel treatment options, there is increasing demand for potential biomarkers for ovarian cancer [71]. The most commonly used biomarker is represented by CA-125. However, it would be worth to identify novel biomarkers for early detection to achieve better survival rates of patients. As miRNAs have been shown previously to be potent biomarkers for ovarian cancer prognosis [21], we analyzed miR-147b-5p which induced apoptosis specifically in ovarian cancer cell lines. MiR-147b is already used as biomarker for survival or prognosis of patients with NSCLC [69] and gastric cancer [72], but also for other diseases like tuberculosis [73]. The overexpression of miR-147b in colon and lung cancer cells causes mesenchymal-to-epithelial transition [74] and plays a role in the resolution phase of inflammation [75]. Our experimental data as well as supporting data from the TCGA showed that hsa-miR-147b might be used as potential biomarker in prognosis of ovarian CA.

Age is suggested to have the strongest impact on ovarian cancer [76]. Hence, we specifically had a closer look on the expression of miR-147b in older patients. The data clearly pointed towards a statistical significant impact on median survival in patients older than 69 years. We observed an increased expression of hsa-miR-147b in tumors of patients with better outcome which suggests the miRNA having a protective effect. Our finding suggests that tumors with decreased expression of hsa-miR-147b might be intrinsically more resistant against standard-of-care or acquire resistance in the course of the treatment. Hsa-miR-147b might be considered as prognostic marker predicting median survival of ovarian cancer. In order to qualify as prognostic marker this miRNA requires validation in an independent matched ovarian cancer cohort in the near future. MicroRNAs in general are good candidates for the development of plasma biomarker due to their stability in blood plasma, ease of isolation and detection, and the characteristic diseases associated expression patterns [77]. They are found to be present in exosomes (exosomal shuttle RNA, esRNA) [78], as well as in Ago2 protein complex fractions in blood plasma (circulating miRNAs, [79]). It has been shown already that circulating miR-147b is present in peripheral blood samples [80].

Modulation of miRNA is a potential tool to improve efficacy of chemotherapy in ovarian CA [81]. First line chemotherapy in ovarian CA most commonly employs carboplatin and paclitaxel at all stages of the disease [7]. However, in recurrent or persistent ovarian CA the antiangiogenetic VEGFA-inhibitor Bevacizumab (Avastin^®^) is administered in combination with cytotoxic taxanes or platinum derivatives [7]. So, effects of the discovered potentially pro-apoptotic miRNAs in combination with the chemotherapeutic drug carboplatin were studied mimicking a more clinically relevant treatment setup. Additive effects of miR-1912-5p, -147b-5p, -96-5p or -3073a-3p in combination with carboplatin in SKOV3, OVCAR3, TOV21G, TOV112D, A2780 and even in the cisplatin resistant cell line A2780-cis suggest a potential benefit in combined application behind the recent treatment with established VEGF-inhibitors. As suggested by others miR-147-family might overcome the cisplatin resistance-associated targets via VEGFA-signaling [81]. Predicted targets for miR-147 include a panel of potential candidates involved in angiogenesis-signaling as Mitogen-activated protein kinase 1 (MAPK1), GTPase NRas (NRAS), Rho GTPase-activating protein 1 (ARHGAP) as well as members of Protein kinase B (AKT) and Protein kinases C family (PRKCA, PRKCB, SDK1). Recently, miR-147b was suggested to regulate endothelial barrier function by targeting expression of the transmembrane metalloprotease ADAM15 involved in cell adhesion and ectodomain processing of cytokines and adhesion molecules [82].

Summarized, our validation screening in three human cancer cell lines as well as the successful verification in ovarian cancer cell lines identified numerous novel pro-apoptotic miRNAs paving the way for the identification of innovative therapeutic entities and for potential biomarkers in the context of ovarian cancer as shown for miR-147b.

## MATERIALS AND METHODS

### Cell culture

SGBS and SKOV3 cells were cultured in DMEM high glucose medium (BioWest, Nuaillé, France) whereas T98G, HCT 116, OVCAR3, TOV21G and TOV112D cells were grown in RPMI-1640 (Life Technologies, Carlsbad, CA, USA). Both media contained 10% (v/v) FBS (Sigma-Aldrich, München, Germany). A2780 and A2780-cis cells were both grown in RPMI-1640 media with 20% (v/v) FBS. All media contained 4 mM stable Glutamin. The cells were grown up to a density of 70% in T75 flasks (Greiner Bio-One, Frickenhausen, Germany) at 37°C and 5% CO_2_. The phenotype of all cell lines was proofed frequently by microscopy. SGBS cells were monitored with differentiation assay [83].

To detect the adherent cell density of the culture the automated single well microscope NyOne (SynenTec Bio Services, Münster, Germany) was used with a magnification of 10×.

### Transfection with miRNA mimics

The cells were seeded 24 h before transfection at 15% confluency in 96-well cell culture plates (Greiner Bio-One). SGBS cells were seeded at a density of 6,000 cells/cm^2^, T98G were seeded at 7,500 cells/cm^2^. HCT 116 and SKOV3 cells were seeded at a density of 10,000 cells/cm^2^. TOV21G, TOV112D as well as A2780 and A2780-cis cells were seeded at a density of 16,000 cells/cm^2^. The cells were transfected with a final concentration of 50 nM miRNA mimic (Qiagen, Hilden, Germany, miRBase Version 21). A non-targeting siRNA (NT) as well as a human cell death control siRNA (DT) (Qiagen) served as functional controls. ScreenFect^®^A reagent (InCella, Eggenstein-Leopoldshafen, Germany) was mixed 1:125 with dilution buffer. A 1 + 1 mix of ScreenFect^®^A and miRNA mimic was incubated for 25 min at room temperature and put on the cells together with 90 μl of fresh medium [25]. The transfected cells were incubated 72 h, in case of cytochrome c and tetramethylrhodamine ethyl ester (TMRE) assay 48 h, at 37°C and 5% CO_2_ before starting analysis.

### Analysis of apoptotic and necrotic cells

Apoptotic cells were identified by flow cytometry measuring the amount of cells with reduced DNA content (sub G0/G1) as previously described by Rudner et al. [84]. In brief, adherent cells were detached from the plate with trypsin (Biochrome, Berlin, Germany). The detachment was stopped with fresh serum-containing medium followed by a centrifugation for 3 min at 130 × g. The cell pellet was resuspended with a isotonic phosphate buffered saline (PBS) buffer containing Triton-X-100 and propidium iodide (PI) (0.1% (w/v) Trisodium citrate, 0.05% (v/v) Triton X-100, 10 μg/ml PI and 3.3 μg/ml RNase A (Life Technologies) in phosphate buffered saline (GE Healthcare, Buckinghamshire, UK)). Cells were incubated for 30 min at room temperature in the dark.

Necrotic cells were identified by flow cytometry of cells stained with PI using a final concentration of 25 μg/ml in RPMI-1640 media in 96-well U-bottom plates with an incubation time of 30 min at 37°C in dark.

The quantification of the staining was done by the MACSQuant^®^ Analyser (Miltenyi Biotec, Bergisch-Glattbach, Germany) equipped with a violet (405 nm), blue (488 nm), and red (635 nm) excitation laser. About 60,000 cells were counted in a 96-well U-bottom plate. The detection was performed with the fluorescence channel B2 for measuring PI exclusion and B2 and B3 for determining the subG0/G1 fraction.

### Detection of mitochondria potential and cytochrome c release

For detection of the mitochondrial potential of the cells a TMRE assay was used. The cells were detached as described and mixed with TMRE in RPMI at a final concentration of 300 nM. 20 min after incubation at 37°C in the incubator the cells were analyzed by flow cytometry using the fluorescence channel B2. 5 μM Carbonylcyanid-m-chlorphenylhydrazon (CCCP) was added as a positive control.

To stain for free cytochrome c after mitochondrial damage the cells were detached as described, fixed with 4% (w/v) Paraformaldehyde for 15 min, permeabilised with 0.1% (v/v) Triton X-100 and stained with an antibody against cytochrome c labelled with Alexa Fluor 488 (BLD-612308; Biozol, Eching, Germany) for 1 h in dark. The antibody was diluted 1:1,500 in BSA. The quantitative analysis was performed with the MACSQuant^®^ Analyser using fluorescence channel B1.

### RT-PCR

The extraction of total RNA was done using the High Pure miRNA Isolation Kit from Roche Diagnostics (Mannheim, Germany). 300 ng of isolated RNA was transcribed via the miScript II RT Kit (Qiagen, Hilden, Germany) using 5× miScript HiSpec Buffer and an incubation time of 60 min at 37°C. The 1:10 diluted cDNA was further analyzed in the Roche Light Cycler 480 using GreenMasterMix (Genaxxon Bioscience, Ulm, Germany). For the qRT-PCR reaction the primers for hsa-miR-96-3p (5′-AATCATGTGCAGTGCCAATATG-3`), hsa-miR-96-5p (5′-TTTGGCACTAGCACATTTTTGCT-3′), hsa-miR-147b-3p (5′-GTGTGCGGAAATG CTTCTG CTA-3′), hsa-miR-147b-5p (5′-TGGAAACATTTCTGC ACAAACTAG-3′), hsa-miR-1912-3p (5′-TACCCAGAG CATGCAGTGTGAAC-3′) and hsa-miR-1912-5p (5′-TGC TCATTGCATGGGCTGTGTA-3′) were used together with the universal reverse primer from the miScript PCR Starter Kit (Qiagen). U6 primer forward (5′-CTCGCTTCGGCA GCACA-3′) and U6 primer reverse (5′-AACGCTTCAC GAATTTGCGT-3′) were used as housekeeping control.

### Western Blotting

For protein analysis the cells were lysed with radioimmunoprecipitation assay buffer , containing 1% (v/v) Nonidet P40, 0.5% (w/v) Sodium-Deoxycholate, 1 mM EDTA and 0.5 μM DTT. After freeze thaw two times the lysate was centrifuged with 4,000 x g for 10 min. The protein concentration in the supernatant was measured with a BCA-assay detecting the OD of the solution at a wave length of 562 nm. 35 μg Protein were loaded on an Amersham ECL Gel 8–16% and run for 1.5 h. The blot was done on a PVDF-membrane (Carl Roth, Karlsruhe, Germany) and run for another 1.5 h. The blocking with a 5% (w/v) BSA-solution in TBST was done for 1 h. The incubation of the primary antibody followed overnight at 4°C. The antibodies against Caspase-3 (cs#9662), cleaved Caspase-3 (cs#9664), PARP (cs#9542), cleaved PARP (cs#9541), cs#5023, cs#12105, cs#2764 from Cell Signaling (Danvers, United States) as well as the anti-Bcl2 (sc-7382) from Santa Cruz (Heidelberg, Germany) were diluted 1:1,000 in blocking solution. The antibody against β-Actin (A5441, Sigma Aldrich) was diluted 1:10,000 in blocking solution and incubated for 1 hour at room temperature. The secondary anti-rabbit IgG, HRP-linked (cs#7074, Cell Signaling) or anti-mouse IgG, HRP linked (A4416, Sigma Aldrich) was diluted 1:10,000 in blocking solution and incubated for 1 h at room temperature. The chemiluminescence signal was detected with the Fusion Fx (Vilber Lourmat, Eberhardzell, Germany) using the appropriate software and the WesternBright ECL HRP substrate (advansta, Palo Alto, USA).

### Caspase activity assay

To detect caspase-3/−7 activities the CellEvent™ Caspase-3/7 Flow Cytometry Assay Kit (Life Technologies, Darmstadt, Germany) was used. The cells were detached as described, resuspended in fresh media and mixed with CellEvent™ Caspase-3/7 detection reagent with a final concentration of 500 nM. 25 min after incubation SYTOX^®^ AADvanced™ was added with a final concentration of 100 μM. The detection was done 5 min after an additional incubation by flow cytometry using the fluorescence channel B1 and B3.

### TCGA-Analysis

In order to characterize the impact of miRNA hsa-miR-147b on median survival in ovarian cancer patients, level 3 miRNA expression data of the “Ovarian serous cystadenocarcinoma” data set were downloaded along with clinical data from the cancer genome atlas (TCGA) data base (www.tcga-data.nci.nih.gov/tcga) using the “firehose-get“ command-line tool (https://confluence.broadinstitute.org/display/GDAC/Download). A detailed description of the clinical characteristics of the cohort can be found in a study by Cancer Genome Atlas Research Network [37]. In total miRNA array data along with the clinical data of 530 patients were matched. For the delineation of the prognostic effect of hsa-miR-147b in different age groups the dataset defined by the first quartile, mean, median and the third quartile of the age distribution (range: 26–89 years, mean: 59.9 years), differential survival analysis for patients with high- versus low-expression of hsa-miR-147b, whilst the median expression used as threshold, was conducted. For this, the log-rank test was applied on the resulting cox-proportional hazard-models and for the purpose of visualization Kaplan-Meier plots were generated.

### Statistical analysis

Data in general were expressed as mean ± SD. Statistical analysis was carried out using GraphPad Prism Version 5.04. Differences of the apoptotic effects were examined by two-way ANOVA followed by Bonferroni pos*t-test*. A *p-value* < 0.05 was considered to be statistically significant.

In case of miRNA screening in the human tumor cell lines, only a selected enriched/censored subpanel of 188 pro-apoptotic miRNAs obtained in initial screening [24] was investigated. Data values were log transformed and normal distribution of the pro-apoptotic effects was analyzed by Kolmogorov-Smirnov test [85].

In order to calculate the Z-score of the screening controls to prove for the validity of the data the triplicates were averaged and the SD was calculated. The Z-score was used as statistical mean to express how many standard deviations are above or below the mean of all measurements.

For TCGA analysis all calculations were conducted using the R statistical platform [86] using functions from the CRAN package survival (www.cran.r-project.org/web/packages/survival).

Furthermore the authors are grateful to Dr. Anne-Marie Mes-Masson from the Centre Hospitalier de l´Université de Montréal (CHUM) for providing us the TOV21G and TOV112D cells for this study.

## SUPPLEMENTARY MATERIALS FIGURES


