# Supplementary material for: Investigation on tissue specific effects of pro-apoptotic micro RNAs revealed miR-147b as a potential biomarker in ovarian cancer prognosis
Source: Oncotarget. 2016 Nov 4;8(12):18773–91. doi: 10.18632/oncotarget.13095 (PMC5386646; doi:10.18632/oncotarget.13095)
Supplement: Supplementary file 1 [file oncotarget-08-18773-s001.pdf]

Investigation on tissue specific effects of pro-apoptotic micro RNAs revealed miR-147b as a potential biomarker in ovarian cancer prognosis

Supplementary Materials

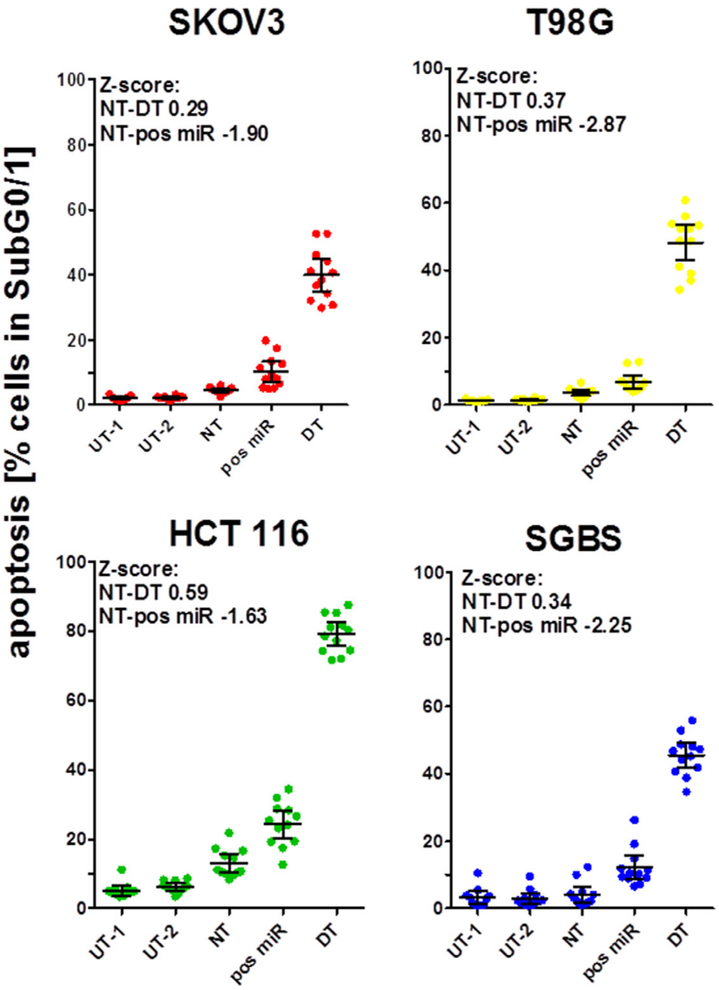

Supplementary Figure S1: Establishment of cell type specific screening controls for transfection efficiency. Untransfected cells with (UT-2) and without transfection reagent (UT-1) were present on each screening plate. In order to normalize the results cells were transfected with a non-targeting miRNA control (NT). To proof efficiency and functionality cells were transfected with either a functional cell death inducing miRNA (pos miR; mmu-miR-137-3p for SKOV3, SGBS and T98G cells; mmu-miR-28a-5p for HCT 116 cells) or with a death control siRNA (DT) (conditions of the experiment as described in Figure 1). The plot represents the median (center line) as well as 25/75% values of corresponding replicates.

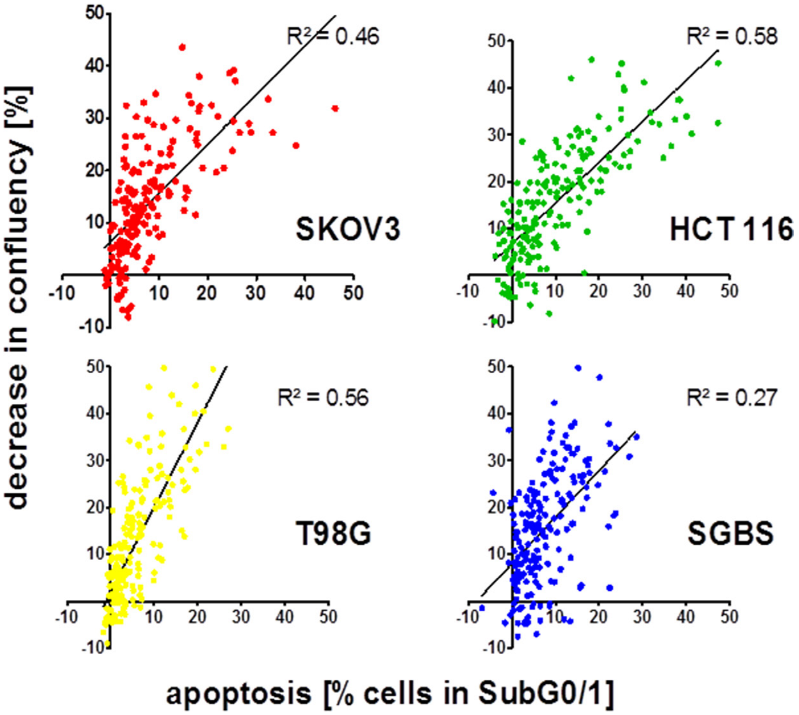

Supplementary Figure S2: Correlation of apoptosis to cell confluence. Correlation between the specific confluency reduction and the specific apoptotic effect under the conditions of the experiment described in Figure 1. The values were normalized by the values of the NT control miRNA. [*n* = 188]
